# Effects of adjective use of melatonin and vitamin C in the treatment of chronic periodontitis: A randomized clinical trial

**DOI:** 10.15171/joddd.2017.041

**Published:** 2017-12-13

**Authors:** Mohammadtaghi Chitsazi, Masoumeh Faramarzie, Mehrnoosh Sadighi, Adileh Shirmohammadi, Arash Hashemzadeh

**Affiliations:** ^1^Department of Periodontics, Faculty of Dentistry, Tabriz University of Medical Sciences, Tabriz, Iran; ^2^Department of Periodontics, Dental and Periodontal Research Center, Tabriz University of Medical Sciences, Tabriz, Iran; ^3^DDS, Private Practice, Tabriz, Iran

**Keywords:** Dental scaling, melatonin, periodontitis, vitamin C

## Abstract

***Background.*** Melatonin can eliminate free radicals and this function can be intensified byvitamin C. Melatonin and vitamin C can have synergic antioxidant effects. The aim of thisstudy was to investigate the effects of adjunctive use of melatonin and vitamin C in the non-surgical treatment of chronic periodontitis.

***Methods.*** Sixty subjects with chronic periodontitis were included in this study and randomly allocated to three groups: group 1) 20 patients received non-surgical periodontal treatment; group 2) 20 patients received non-surgical periodontal treatment with adjunctive use of melatonin; and group3) 20 patients received non-surgical periodontal treatment with combination use of melatonin + vitamin C. Clinical parameters (PD, CAL,GI) were recorded at baseline and at 3-month and 6-month intervalsafter treatment. Data were analyzed with paired t-test, one-way ANOVA andrepeated-measures ANOVA. A significant difference was assumed at P<0.05.

***Results.*** Non-surgical periodontal therapy improved PD and CAL 3 and 6 months treatment compared to baseline (P<0.001). There was a significant improvement in PD and CAL scores at 6-month interval compared to 3 months in the melatonin+ vitamin C group (P<0.05), while the differences in PD and CAL scores between the mentioned intervals were not significant between the control and melatonin groups (P>0.05). Therefore adjunctive dose of vitamin C offered an additional effect at this interval.

***Conclusion.*** Combination therapy with melatonin and vitamin C can improve the results of non-surgical periodontal therapy.

## Introduction

Melatonin is a neuroendocrine hormone which is released by the hypophysis with a circadian rhythm and a higher blood level at night.^1,2^ Melatonin level is measured in saliva and plasma.^3^ Melatonin plays not only an important antioxidant role, but also an immune modulatory role. Also, its anti-neoplastic and protective characteristics are of great importance.Melatonin encourages type І collagen and bone synthesis, so it may play a therapeutic role in mechanical, bacterial, viral and fungal injuries of the oral cavity.^4^ Melatonin is a free radical scavenger in both pharmacological and physiologic blood concentrations.^5-7^ Anti-inflammatory effect of melatonin is used in the treatment of periodontal diseases. Periodontal tissue breakdown occurs in response to bacterial plaque and toxic products resulting from host‒microbe interactions. An important feature of periodontitis is production of free oxygen radicals which are released by bacteria and also, host immune response. An imbalance between anti-oxidants and pro-oxidants may lead to a remarkable tissue breakdown.^8^ Gender does not influence the levels of melatonin, whereas factors such as smoking, use of alcohol and aging reduce salivary melatonin levels.^9^ Cutando et al^2^ demonstrated that salivary melatonin levels in patient with periodontitis are lower than healthy subjects. They found that melatonin levels of plasma and saliva increase in diabetic patients with remarkable destruction. They suggested that melatonin increase during diabetes may have a protective role in the periodontium.

Vitamin C has been suggested as a host modulatory agent in periodontal treatment.^10,11^ Vitamin Cdeficiency does not lead to periodontitis but benefits of additional vitamin C have been shown in tissue regeneration and treatment of infectious diseases.^12,13^ Vitamin C deficiency is associated with poor collagen synthesis, impaired wound healing and vascular rupture.^14^ Collagen regeneration is essential for the integrity of tooth attachment apparatus and periodontal health. Vitamin C is important in the synthesis of intercellular substance like collagen fibers;^15^ it also has an immune modulatory function.^16,17^ Therefore serum vitamin C deficiency can be a risk factor for periodontal diseases.^11^

Since an imbalance between free radicals and oxidants can play a major role in chronic periodontitis and given the role of melatonin in the elimination of free radicals and synergic function of vitamin C and melatonin,^18^ the aim of the present research was to investigate the effects of simultaneous use of melatonin and vitamin C in the non-surgical treatment of chronic periodontitis.

## Methods

A total of 60 subjects (29 males and 31 females, aged 23‒65 years; mean age: 41 years) were recruited for a single-masked study from patients referred to the Department of Periodontics, Faculty of Dentistry, Tabriz University of Medical Sciences, over a period of 2 years (April 2014 to March 2016). All the subjects were systemically healthy. Subjects were excluded from the study if 1) they had a prior use of non-steroidal anti-inflammatory drugs or antimicrobial drugs in the last 3 months before the study began; 2) they were lactating or pregnant; 3) they had used mouthwashes or vitamin supplements in the last 3 months before the research; 4) they had a history of current or previous smoking or recreational drug use; and 5) they had special dietary requirements. Sixty volunteers with moderate-to-severe chronic periodontitis, each with at least three 5–7-mm-deep pockets were selected for this study. The subjects were categorized into three groups. Twenty subjects received non-surgical periodontal treatment (SRP group); 20 subjects received non-surgical periodontal treatment with adjunctive use of melatonin (melatonin group); and 20 subjects received non-surgical periodontal treatment with combined use of melatonin and vitamin C (melatonin+ vitamin C group).

The subjects completed medical and dental questionnaires and the study protocol was approved by the Ethics Committee of Tabriz University of Medical Sciences, Tabriz, Iran. After enrollment, all the subjects were instructed in toothbrushing with modified bass technique and also flossing twice a day and then the groups were randomly further divided into three groups using a randomization software. All the patients in the three groups underwent careful SRP by using an ultrasonic device (Various 350, NSK, Japan) and standard Gracey periodontal curettes (Hu-Fridey Instruments, Chicago, IL, USA). Periodontal therapy was conducted by a single periodontist (MC). The melatonin group (n=20) underwent a protocol of conventional non-surgical treatment with an adjunctive dose of melatonin (2 mg a day for 4 weeks). The melatonin + vitamin C group (n=20) received non-surgical treatment with adjunctive dose of melatonin (2 mg a day for 4 weeks) and vitamin C (60 mg for females and 75 mg for males for 4 weeks). The patients were recalled 3 and 6 months after treatment to record the clinical measures (GI, CAL and PD) again.

Clinical measurements were made with a standard periodontal probe used for periodontal probing (UNC-15, Hu-Fridey Instruments, Chicago, IL, USA). Clinical examinations were carried out by a single examiner (MF) who was an experienced periodontist and blinded to the type of treatment. Clinical indices were obtained at four surfaces per tooth: mesiofacial, buccal, distofacial and lingual. PD and CAL were recorded in duplicate, and when the difference between the measurements was 1 mm, a third evaluation was carried out and the mean measurement of each site was calculated from the closest two of the triplicate probing measures. Gingival index (GI) was also assessed.

### Statistical analysis

The SPSS 14.0 was used for analysis of data, with paired t-test, one-way ANOVA and repeated-measures ANOVA. Statistical significance was set at P<0.05.

## Results

The mean GI of the study groups at baseline and 3 and 6 months after treatment are presented in Table 1. There were a significant improvement in GI after 3 and 6 months in both melatonin and melatonin+ vitamin C groups compared to baseline (P<0.001). However, the differences between the 6-month and 3-month intervals were not significant (P>0.05).

**Table 1:** Comparison of GI at different observation periods in different groups

**Clinical parameter**	**Evaluation**	**Control**	**Melatonin**	**Melatonin+ Vit C**	**Inter-group P-value**
**GI**	Δ 0-3 M	0.45±0.13	0.54±0.12	0.63±0.11	0.95
	Intra-group P value	P<0.001	P<0.001	P<0.001	
	Δ 0-6 M	0.63±0.24	0.83±0.21	0.92±0.22	0.08
	Intra-group P value	P<0.001	P<0.001	P<0.001	
	Δ 3-6 M	0.33±0.22	0.62±0.14	0.75±0.13	0.12
	Intra-group P value	P=0.479	P=0.401	P=0.309	

The mean PD and CAL of the study groups at baseline and at 3-month and 6-month post-operative intervals are presented in Tables 2 and 3.

**Table 2:** Comparison of PD at different observation periods in different groups

**Clinical parameter**	**Evaluation**	**Control**	**Melatonin**	**Melatonin+ Vit C**	**P-value**
**PD (mm)**	Baseline	6.40±1.20	6.41±1.02	6.43±1.17	0.031
	3 months	5.23±1.89	4.56±1.31	4.41±1.15	
	P-value	P<0.001	P<0.001	P<0.001	
	Δ 0-3 M	1.17 ± 0.41	1.85 ± 0.70	2.02±0.41	
	Baseline	6.40±1.20	6.41±1.02	6.43±1.17	0.028
	6 months	4.92±1.53	3.54±1.45	3.08±1.12	
	P-value	P<0.001	P<0.001	P<0.001	
	Δ 0-6 M	1.48±0.40	2.87±0.69	3.35±0.64	
	3 months	5.23±1.89	4.56±1.31	4.41±1.15	0.08
	6 months	4.92±1.53	3.54±1.45	3.08±1.12	
	P-value	P=0.325	P=0.141	P=0.035	
	Δ 3-6 M	0.13±0.18	1.02±0.33	1.33±0.12	

**Table 3:** Comparison of CAL at different observation periods in different groups

**Clinical parameter**	**Evaluation**	**Control**	**Melatonin**	**Melatonin + Vit C**	**P value**
**CAL (mm)**	Baseline	6.23±1.22	6.29±1.16	6.30±1.21	0.026
	3 months	5.14±1.23	4.23±1.43	4.12±1.95	
	P value	P<0.001	P<0.001	P<0.001	
	Δ 0-3 M	1.09±0.53	2.06±0.47	2.18±0.55	
	Baseline	6.23±1.22	6.29±1.16	6.30±1.21	0.018
	6 months	4.56±1.16	3.22±1.52	3.00±1.53	
	P value	P<0.001	P<0.001	P<0.001	
	Δ 0-6 M	1.67±0.61	3.07±0.55	3.30±0.66	
	3 months	5.14±1.23	4.23±1.43	4.12±1.95	0.075
	6 months	4.56±1.16	3.22±1.52	3.00±1.53	
	P value	P=0.104	P=0.182	P=0.042	
	Δ 3-6 M	0.58±0.13	1.01±0.41	1.12±0.34	

Non-surgical periodontal treatment improved PD and CAL at 3-month and 6-month postoperative intervals compared to baseline (P<0.001). There were significant improvements in PD and CAL scores at 6-month postoperative interval compared to 3-month interval in the melatonin + vitamin C group (P=0.035 and P=0.042, respectively), while the differences in PD and CAL scores in the mentioned intervals were not significant between the control and melatonin groups (P>0.05). Therefore the adjunctive dose of vitamin C offered an additional effect at this interval.

## Discussion

The periodontal disease is an inflammatory process in which the bone and periodontal ligament are destroyed via a restorative process by osteoclasts. Cytokines and local factors secreted by host defense cells in response to bacterial attack mediate this process. Melatonin plays a critical role in the regulation of this protein-mediated process.^19^ The aim of this research was to study the effect of adjunctive use of melatonin and vitamin C in the non-surgical treatment of patients with chronic periodontitis.

In the present study we found that the combined use of vitamin C and melatonin supplements with non-surgical periodontal therapy in periodontitis with more than 5 mm pockets significantly reduced the amount of PD and CAL compared to scaling alone. Improvements in these clinical parameters following non-surgical periodontal treatment are parallel to the previous findings in this regard but the differences in the scores can be attributed to the adjunctive use of melatonin and vitamin C.

Cutandoet al^20^ reported the same results in diabetic patients with chronic periodontitis. They demonstrated significant improvements in clinical parameters (PD, CAL and GI) in the study group with adjunctive melatonin use. The greater improvements in a study by Cutandoet al^20^ compared to present study can be attributed to the local use of melatonin.

Animal studies and clinical trials have documented therapeutic effects of melatonin.^20-23^ Systemic and local use of melatonin in rats with lipopolysaccharide-induced periodontitis reduced serum levels of aspartate aminotransferase and alanine transaminase significantly compared to the control group.^24,25^ These studies suggest the adjunctive use of melatonin in the treatment of chronic periodontitis.

In the current study, a combination of melatonin and vitamin C as an adjunctive therapy was used; improvements in clinical parameters were greater in the melatonin+vitamin C group in comparison to the other two groups. In addition, at 6-month postoperative interval differences in PD and CAL scores were statistically significant compared to 3-month interval while the difference in the same interval in PD and CAL were not statistically significant in the two other groups. This can be attributed to the long-term effects of combination therapy with melatonin and vitamin C as a host-modulatory agent but the role of vitamin C deficiency in periodontitis isunknown.^26^ Vitamin C deficiency does not lead to periodontitis but it has been proved that additional dose of vitamin C is necessary during tissue regeneration and treatment of infectious diseases.^27^ Vitamin C plays an important role in collagen synthesis found in intracellular matrix and also in tooth and bone matrix.

Abou Sulaiman et al^28^ reported that adjunctive use of vitamin C did not improve the clinical parameters compared to non-surgical periodontal treatment alone. Also, Leggott et al^29^ did not find any benefits in the additional use of vitamin C supplements in terms of PD and BOP. The positive effect of vitamin C in our study can be attributed to the synergic effect of melatonin and vitamin C.^20^ As mentioned above, before vitamin C could be a pre-oxidant under certain conditions.^30^ According to Gitto’s^18^ study, when melatonin is combined at a concentration lower than its effective dose with pro-oxidant concentration of vitamin C, it exhibits a higher anti-oxidative function.^18^ This synergistic function increased with an increase in the dose of melatonin. The reason for this increase in synergic function may be the fact that vitamin C recycles melatonin.^31^ if so, this could explain the enhanced effects of melatonin with vitamin C.

## Conclusion

Based on the results of the present study, treatment with melatonin and vitamin C as an adjunct to SRP may improve periodontal indexes (PD, CAL, and GI) compared to SRP alone. Combination of these two supplements showed better effects in the long term.

## Acknowledgments

The authors would like to acknowledge the Dental and Periodontal Research Center at Tabriz University of Medical Sciences for the financial support of this project.

## Authors’ contributions

The study was planned by MC and MH. The statistical analyses and interpretation of data were carried out by MS. MC, MF, AH and AS contributed to the literature review. All the authors have read and approved the final manuscript.

## Funding

The study was supported by Dental and Periodontal Research Center at Faculty of Dentistry, Tabriz University of Medical Sciences.

## Competing interests

The authors declare that they have no competing interests with regards to authorship and/or publications of this paper.

## Ethics approval

The study protocol was approved by the Ethics Committee of Tabriz University of Medical Sciences.
